# Early treatment response assessment with [^177^Lu]PSMA whole-body-scintigraphy compared to interim PSMA-PET

**DOI:** 10.1186/s40644-024-00773-w

**Published:** 2024-09-19

**Authors:** David Ventura, Philipp Rassek, Philipp Schindler, Burak Han Akkurt, Linus Bredensteiner, Martin Bögemann, Katrin Schlack, Robert Seifert, Michael Schäfers, Wolfgang Roll, Kambiz Rahbar

**Affiliations:** 1 Department of Nuclear Medicine, University Hospital Münster, Albert-Schweitzer-Campus 1, 48149, Münster, Germany; 2 West German Cancer Center (WTZ), 48149, Münster site, Germany; 3 Department of Radiology, University Hospital Münster, 48149, Münster, Germany; 4 Department of Urology, University Hospital Münster, 48149, Münster, Germany; 5 Department of Nuclear Medicine, University Hospital Bern, 3010, Bern, Switzerland; 6 European Institute for Molecular Imaging (EIMI), University of Münster, 48149, Münster, Germany

**Keywords:** [^177^Lu]PSMA, Radioligand therapy, WBS, PSMA-PET, mCRPC

## Abstract

**Background:**

Prostate-specific membrane antigen positron emission tomography (PSMA-PET) is an essential tool for patient selection before radioligand therapy (RLT). Interim-staging with PSMA-PET during RLT allows for therapy monitoring. However, its added value over post-treatment imaging is poorly elucidated. The aim of this study was to compare early treatment response assessed by post-therapeutic whole-body scans (WBS) with interim-staging by PSMA-PET after 2 cycles in order to prognosticate OS.

**Methods:**

Men with metastasized castration-resistant PC (mCRPC) who had received at least two cycles of RLT, and interim PSMA-PET were evaluated retrospectively. PROMISE V2 framework was used to categorize PSMA expression and assess response to treatment. Response was defined as either disease control rate (DCR) for responders or progression for non-responders.

**Results:**

A total of 188 men with mCRPC who underwent RLT between February 2015 and December 2021 were included. The comparison of different imaging modalities revealed a strong and significant correlation with Cramer V test: e.g. response on WBS during second cycle compared to interim PET after two cycles of RLT (c_φ_ = 0.888, *P* < 0.001, *n* = 188). The median follow-up time was 14.7 months (range: 3–63 months; 125 deaths occurred). Median overall survival (OS) time was 14.5 months (95% CI: 11.9–15.9). In terms of OS analysis, early progression during therapy revealed a significantly higher likelihood of death: e.g. second cycle WBS (15 vs. 25 months, *P* < 0.001) with a HR of 2.81 (*P* < 0.001) or at PET timepoint after 2 cycles of RLT (11 vs. 24 months, *P* < 0.001) with a HR of 3.5 (*P* < 0.001). For early biochemical response, a PSA decline of at least 50% after two cycles of RLT indicates a significantly lower likelihood of death (26 vs. 17 months, *P* < 0.001) with a HR of 0.5 (*P* < 0.001).

**Conclusion:**

Response assessment of RLT by WBS and interim PET after two cycles of RLT have high congruence and can identify patients at risk of poor outcome. This indicates that interim PET might be omitted for response assessment, but future trials corroborating these findings are warranted.

**Supplementary Information:**

The online version contains supplementary material available at 10.1186/s40644-024-00773-w.

## Background

Prostate cancer (PC) is the second most common malignancy in men, with an estimated 1.4 million new cases worldwide each year [1]. Patients with localized hormone sensitive disease have a good prognosis compared to patients with metastatic castration-resistant PC (mCRPC) [2, 3]. However, the prognosis of mCRPC has improved in recent years due to new therapeutic approaches [4, 5]. Immunotherapy or genomic-based therapies may have a positive impact in selected subgroups of mCRPC [6, 7]. Targeted α-therapy using [^223^Ra]radium-dichloride has been shown to be a safe treatment modality with increased overall survival (OS) in metastatic bone-dominant disease [8, 9]. The VISION trial demonstrated that radioligand therapy (RLT) using the prostate specific membrane antigen (PSMA) labelled with the β-emitter [^177^Lu]lutetium ([^177^Lu]PSMA) significantly improved imaging-based progression-free survival (PFS) and OS [10].

In recent years, radiolabeled PSMA using positron emission tomography (PET) has become a routinely used imaging method in the diagnosis and treatment of PC [11]. The impact of presurgical staging and biochemical recurrence on patients has been thoroughly established [12, 13]. A benefit of PSMA-PET scan over morphological imaging was also shown in metastatic PC [14]. For evaluation of RLT eligibility, patients must undergo PSMA imaging to assess PSMA expression [15]. Therefore, the new PROMISE V2 framework recommended PSMA expression to be at least equal to or above the level of physiological liver uptake [16]. According to current SNMMI/EANM guidelines, interim PSMA-PET scans should be conducted every 12 weeks during PSMA therapy to ensure optimal follow-up and treatment response assessment [10, 15]. However, resources for serial PSMA-PET scans are often limited in a clinical setting. Furthermore, the role of PSMA-PET in monitoring the treatment of mCRPC is less clear. The Prostate Cancer Working Group Criteria 3 (PCWG3) guidelines recommend using conventional computed tomography (CT) and bone scans for treatment monitoring in mCRPC, as performed in the VISION trial, which is rooted in a lack of data on PSMA-targeted imaging for response assessment [10, 17].

Following the initiation of RLT, guidelines recommend assessing prostate-specific antigen (PSA) levels at each cycle to evaluate biochemical response [15]. Additionally, planar whole-body scintigraphy (WBS) should be performed 1–2 days after RLT administration to assess the tumoral PSMA uptake [15]. Some studies have already shown that post-treatment single photon emission computed tomography (SPECT) quantification can help prognosticating PFS [18, 19]. Neubauer et al. (2023) recently reported that early response monitoring, using quantitated post-therapy SPECT scans, prognosticates OS in RLT [20]. However, there is a lack of evidence for WBS as a stand-alone imaging modality to guide response to RLT.

The aim of this study was to evaluate early treatment response for the purpose of predicting OS in a manner that is applicable to clinical practice. This was assessed by post-therapeutic WBS after the second and third cycles compared to established parameters such as PSA response and interim PSMA-PET after two cycles of RLT.

## Methods

### Study design and patient selection

This single-center study was performed as a retrospective observational study at a tertiary care academic medical center. The following criteria were applied to determine the study population: (a) mCRPC with tumoral PSMA expression above liver uptake in PSMA-PET; (b) approval of the interdisciplinary tumor board for RLT with [^177^Lu]PSMA-617; (c) patients received at least 2 cycles of RLT as well as restaging after the second cycle; (d) applicable medical records and imaging follow up; (e) age > 18 years. This study was approved by the local ethics committee (No. 2019–711-f-S). This study has been carried out in accordance with the ethical standards outlined in the 1964 Declaration of Helsinki and its subsequent revisions.

**Fig. 1:**
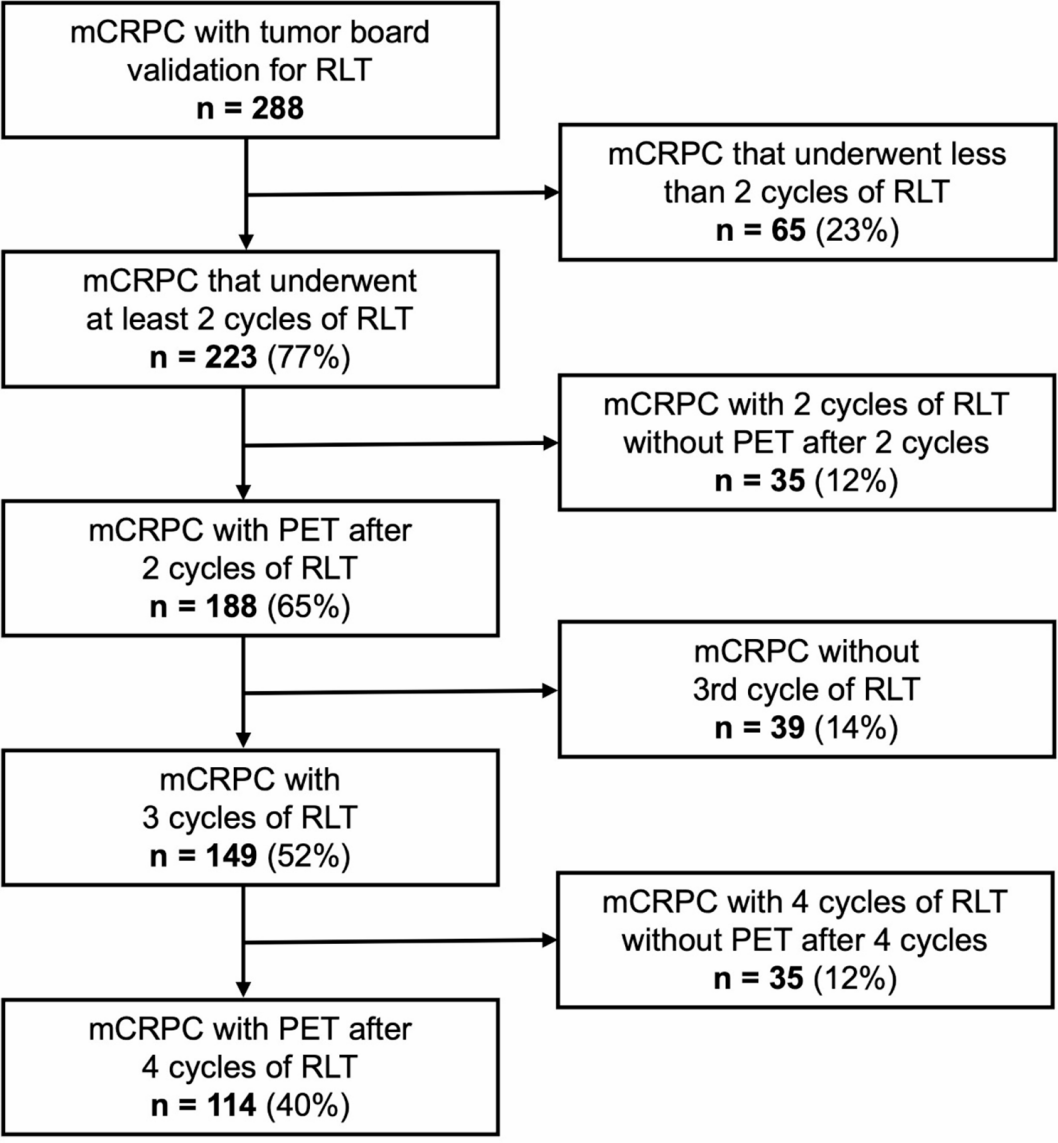
Patient selection process

A total of 288 consecutive patients between 2015 and 2021 (to ensure sufficient follow-up time) underwent [^177^Lu]PSMA-617 therapy. 188 of 288 patients (65%) met the aforementioned inclusion criteria. The flow chart in Fig. 1 displays a comprehensive patient selection process.

### PSMA-PET imaging

All patients underwent serial imaging with either PSMA-PET computed tomography (PET-CT) or magnetic resonance imaging (PET-MRI). PET imaging was performed prior to RLT (initial PET), 6–8 weeks after the second cycle, and 6–8 weeks after the fourth cycle. Examination was performed using either a Biograph mCT 128 or a 3T Biograph mMR system (Siemens Healthcare, Erlangen, Germany). Patients were imaged with inhouse produced radiolabeled [^68^Ga]PSMA-11 or [^18^F]PSMA-1007 as described before [21, 22]. For a valid comparison, patients underwent serial imaging using either [^68^Ga]PSMA-11 or [^18^F]PSMA-1007.

### [177Lu]PSMA-617 and WBS imaging

All patients received [^177^Lu]PSMA-617 (ABX GmbH, Radeberg, Germany) based on literature recommendations [23]. The radiosynthesis procedure and quality control parameters were conducted in-house as previously described [24]. [^177^Lu]PSMA-617 was slowly administered intravenously over a period of 30 s in our therapy unit.

A planar WBS (anterior / posterior) was obtained for each cycle 48 h after injection using a Discovery NM/CT 670 Pro System (General Electric Company Boston, Massachusetts, USA) or a Symbia T2 (Siemens Healthcare, Erlangen, Germany). Image acquisition was performed with parallel collimators in a continuous scanning mode with a zoom of 1.0, utilizing a 2.21 mm pixel size and a speed rate of 15 cm/min with a photopeak at 113.0 and 208.0 keV (± 10%).

### Image analysis

All imaging data were reviewed by two experienced nuclear medicine physicians in consensus. Both reviewers were blinded to the clinical outcome as well as other collected parameters (i.e., PSA progression / regression).

The images were visually interpreted based on the uptake of the radiotracer. For this purpose, the lesional uptake was categorized according to the PROMISE V2 criteria as follows: (0) equal to or lower as blood pool; (1) equal to or lower than liver and higher than blood pool; (2) equal to or lower than parotid gland and higher than liver; (3) higher than parotid gland. In the case of [^18^F]PSMA-1007 with high liver excretion, the spleen was employed as the reference organ in accordance to PROMISE V2 [16]. This assessment included the maximum intensity projection (MIP) of the initial PET and the PET after two cycles of RLT, as well as planar WBS after first, second and third cycle, if applicable. The PROMISE V2 PSMA-expression score was reported for the entire tumor burden (overall), as well as for the lesions with the highest and lowest uptake.

PROMISE V2-scores were correlated between initial PET and first cycle WBS, as well as between third cycle WBS and PET after the second cycle.

### Response assessment

WBS after first, second and third cycle, and restaging PET scans after two and four cycles were compared to initial PSMA-PET. Response was defined as follows: complete response (CR) was defined as the absence of PSMA uptake in all lesions that were previously avid. Partial response (PR) was defined as the absence of PSMA uptake in more than two previously avid lesions or obviously reduced PSMA-avid tumor volume. Progressive disease (PD) was defined as the occurrence of new PSMA-avid lesions or obviously progressed PSMA-avid tumor volume. Stable disease (SD) was defined as neither aforementioned CR, PR, or PD criteria [16]. Disease control rate (DCR) for imaging response assessment was defined as CR, PR, or SD and progress as PD.

The imaging response correlation was used to compare the two different modalities (PET and WBS) in terms of DCR and progression. A correlation analysis was performed between PET after two cycles of RLT and the second cycle WBS, as well as a correlation between the PET after four cycles of therapy and third cycle WBS.

A decline of at least 50% in their PSA levels is considered as biochemical response (PSA50), in line with the previously published studies on mCRPC patients undergoing RLT with [^177^Lu]PSMA-617 using a cutoff of 50% PSA-decline [10]. A correlation analysis was conducted to investigate on the relationship between early biochemical response and imaging response. The analysis involved the PET after two cycles of RLT / WBS of the third cycle and PSA50.

### Statistical and survival analysis

Clinical and demographic data are presented as total number, median, percentage, range and 95% confidence interval (95%CI). Binary and ordinary variables were correlated with Cramer V (c_φ_) and Spearman (r_s_) test. Values of greater than 0.20, 0.40, 0.60 and 0.80 for c_φ_ and r_s_ correspond to low, intermediate, strong, and very strong positive correlation. OS analyses were tested for their prognostic value with log rank test and Kaplan-Meier survival analysis. Hazard ratios (HR) with 95%CI were calculated using a stratified Cox proportional hazard model. Null hypothesis was rejected if *P*-value was less than 0.05 (two-sided for correlation tests). Statistical analysis was performed using SPSS Statistics version 28.0.1.1. (SPSS Inc.).

## Results

### Patients’ characteristics

A total of 188 men (median age: 72 y, range: 44–89) received 898 cycles of RLT with a median of 4 cycles per patient (range: 2–13). The median activity per cycle was 7.2 GBq (range: 5.1–7.8 GBq) and the median cumulative activity for each patient was 36.9 GBq (95%CI: 34.3–39.6 GBq). The median time from initial PET to the first cycle was 40.8 days (95%CI: 37.1–44.5). Between first interim PET after the second cycle of therapy, to the third cycle the median time was 14.7 days (95%CI: 12.5–17.1), respectively. Detailed patients’ characteristics are presented in Table 1.

**Table 1:** Patients’ characteristics

Characteristic		*n*	%	median	range
**Men**		188			
**Age**					
Initial Diagnosis				63	42–87
RLT*				72	44–89
**Gleason Score**					
Gleason 6		9	4.8		
Gleason 7 a / b		62	33.0		
Gleason 8		53	28.2		
Gleason 9		58	30.9		
Gleason 10		6	3.2		
**Pretreatments**					
Prostatectomy		116	61.7		
Prostate radiation		85	45.2		
ADT*		188	100		
Docetaxel		156	83.0		
Cabazitaxel		54	28.7		
Abiraterone		169	89.9		
Enzalutamide		169	89.9		
[^223^Ra]radium-dichloride		35	18.6		
**Disease localization**					
Lymph node metastases		154	81.9		
Distant metastases		175	93.1		
	Bone	173	98.6		
	Liver	37	21.1		
	Lung	31	17.7		
	Brain	6	3.4		
ECOG* performance status					
ECOG 0		56	29.6		
ECOG 1		99	52.4		
ECOG 2		33	17.5		
RLT* activity [GBq]					
1st cycle				7.2	5.2–7.8
2nd cycle				7.2	4.9–7.9
3rd cycle				7.2	4.3–7.8
4th cycle				7.2	4.5–7.7
PSA* [ng/mL]					
Pretreatment				187.5	0.05–12,500
End of treatment				84.2	0.04–7600

*RLT = radioligand therapy; ADT = anti-androgen therapy; ECOG = Eastern cooperative oncology group performance status; PSA = prostate specific antigen

**Fig. 2:**
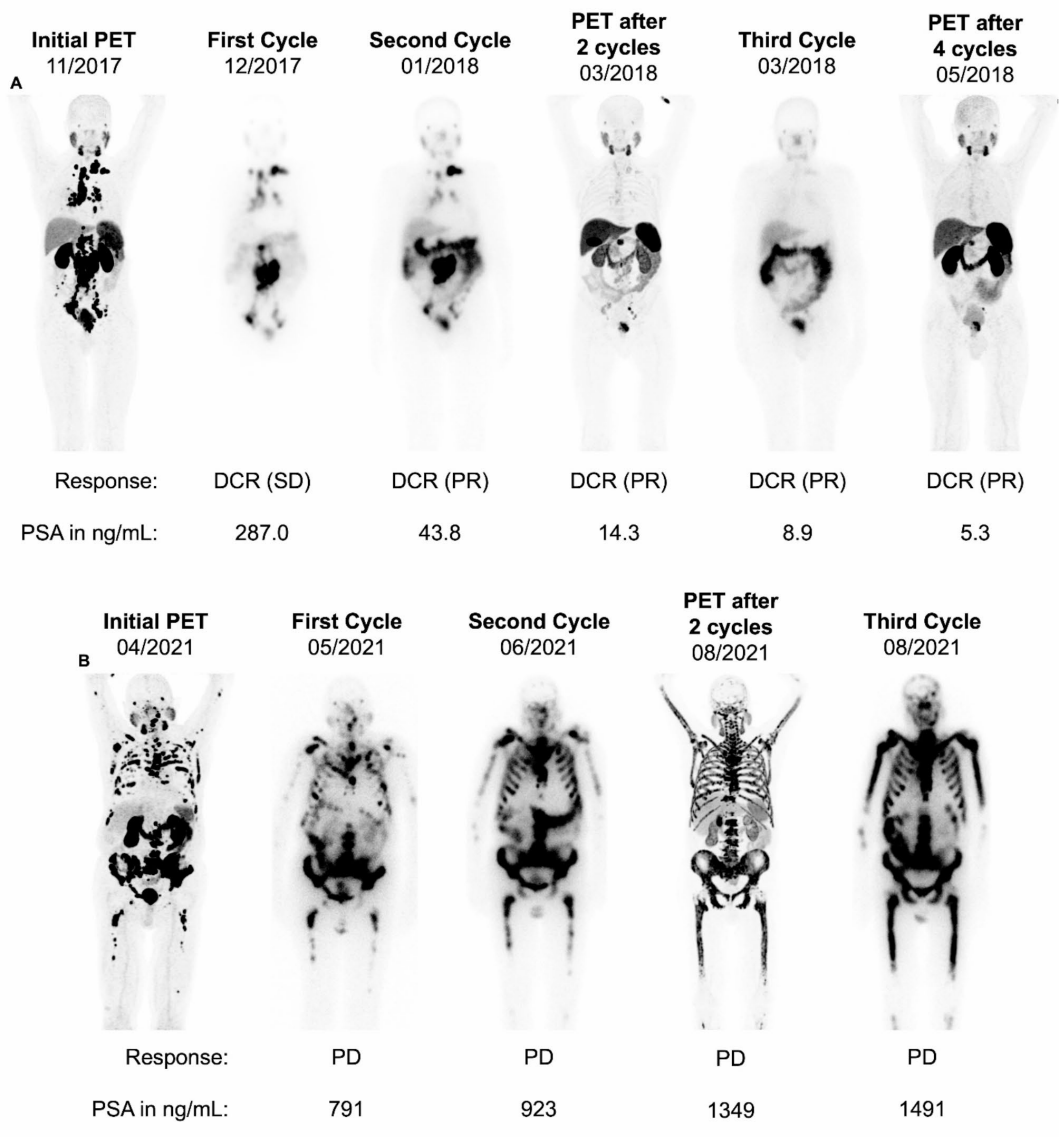
Demonstration of response assessment to RLT. The first cycle WBS was employed for the purpose of comparison and validation of tracer uptake. 73-year-old man with intense lesional PSMA expression (PROMISE V2 score: 3 on initial PET and WBS) in lymph node and bone metastases. This patient had an excellent response to RLT, demonstrating a remarkable decrease in PSMA expression and PSA decline after 4 cycles of RLT (**A**). 76-year-old man with multifocal bone-dominant disease and intense lesional PSMA expression (PROMISE V2 score: 3 on initial PET and WBS). This patient did not respond to RLT and had a progressive, disseminated disease, as evidenced at an early stage by the second cycle of WBS (**B**)

All patients were heavily pretreated and received at least one line of taxane-based chemotherapy and one next generation antihormonal therapy. The median follow-up time was 14.7 months (range: 3–63). During the observation period, 128 patients (68.1%) demonstrated molecular progression based on PSMA-PET-imaging, and 125 patients (66.5%) passed away. At the end of the treatment period, 81 patients (43.1%) demonstrated a 50% decline in PSA levels. Thus, any end of treatment PSA and alkaline phosphatase (ALP) decline occurred in 117 (62.2%) and 102 (54.3%) of cases, respectively. Figure 2 illustrates one example of a patient with DCR (A) and another with PD (B) to RLT.

### PROMISE V2 score and response correlation

The correlation of the PROMISE V2 expression score was used to compare the two different imaging modalities in terms of PSMA uptake. For instance, the correlation coefficient (r_s_) between the overall PROMISE V2 expression score of the initial PET and WBS of the first cycle was 0.813 (*P* < 0.001, *n* = 188). Similarly, for the PET after two cycles of RLT and WBS of the third cycle, it was 0.633 (*P* < 0.001, *n* = 149). The PROMISE V2 score’s detailed findings are presented in Table 2 and Table 1S.

**Table 2:** PROMISE V2 overall expression score

PROMISE V2 expression score	Initial PET (*n* = 188)	First cycle WBS (*n* = 188)	Second cycle WBS (*n* = 188)	PET after two cycles of RLT (*n* = 188)	Third cycle WBS (*n* = 149)
**0**	0	0	0	0	0
**1**	0	1 (0.5)	3 (1.6)	6 (3.2)	4 (2.7)
**2**	42 (23.3)	43 (22.9)	52 (27.7)	75 (39.9)	61 (40.9)
**3**	146 (77.7)	144 (76.6)	133 (70.7)	107 (56.9)	84 (56.4)

Correlation analysis between early biochemical response and imaging response indicated an intermediate correlation: the correlation coefficient (c_φ_) for the PET after two cycles of RLT and PSA50 was 0.406 (*P* < 0.001), and for the WBS of the third cycle and PSA50 it was 0.440 (*P* < 0.001).

Between the two imaging modalities, PET and WBS, the Cramer V test showed a strong and significant correlation for the assessment of response (DCR vs. PD). For instance, the correlation coefficient (c_φ_) between the WBS of the second cycle and the PET after two cycles of RLT was 0.888 (*P* < 0.001, *n* = 188). Similarly, for the WBS of the third cycle to the PET after four cycles, it was 0.803 (*P* < 0.001, *n* = 114). The detailed findings of the imaging response assessment are presented in Table 3.

**Table 3:** Response assessment

Response	Second cycle WBS (*n* = 188)	PET after two cycles of RLT (*n* = 188)	Third cycle WBS (*n* = 149)	PET after four cycles of RLT (*n* = 114)
**Progressive**	72 (38.3%)	66 (35.1%)	49 (32.9%)	31 (27.2%)
**DCR**	116 (61.7%)	122 (64.9%)	100 (67.1%)	83 (72.8%)
*Partial Response + Complete Response*	*53 (28.2%)*	*62 (33.5%)*	*57 (38.2%)*	*55 (48.2%)*
*Stable*	*63 (33.5%)*	*59 (31.4%)*	*43 (28.9%)*	*28 (24.6%)*

### Survival analysis

**Fig. 3:**
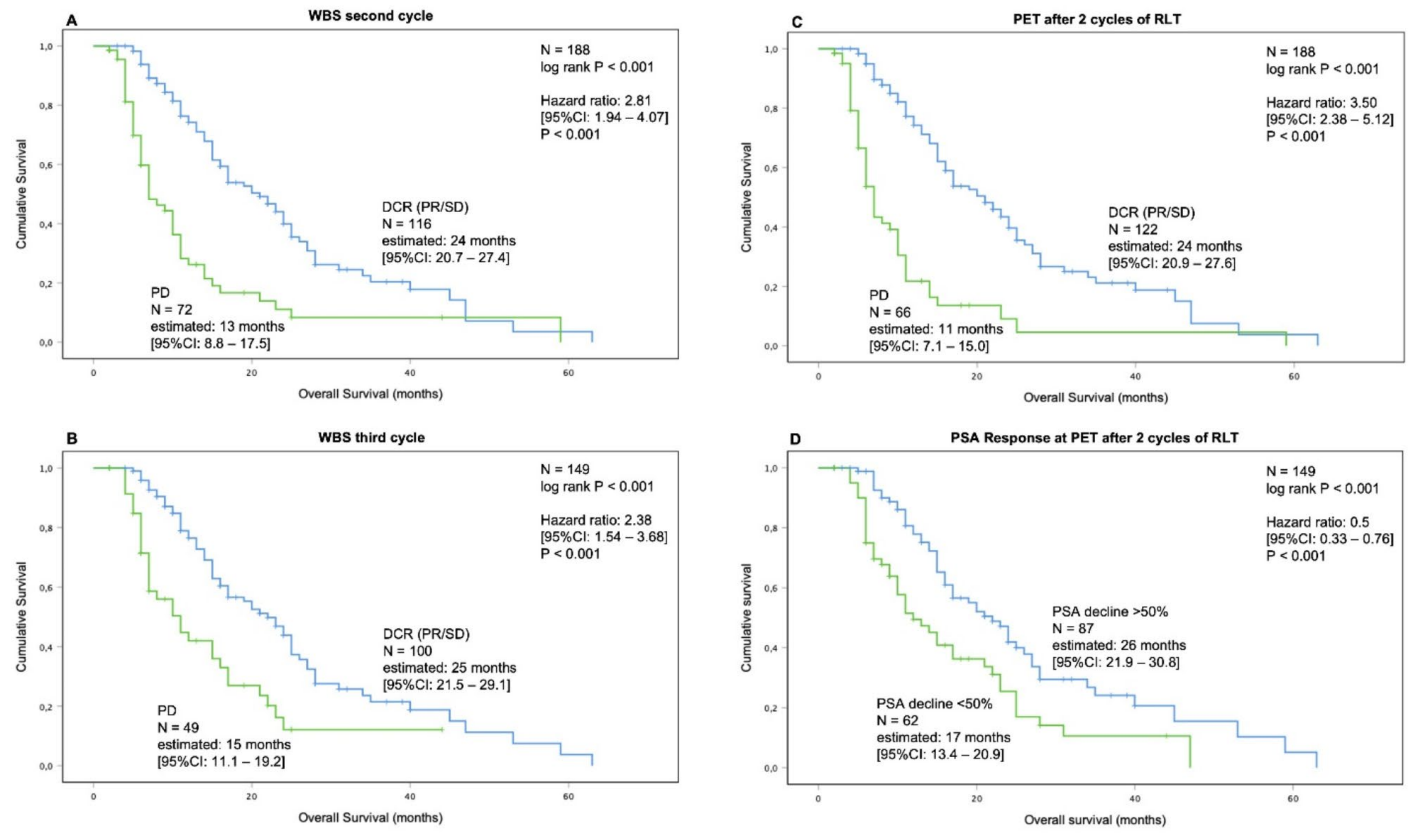
Overall survival analysis. The Kaplan-Meier analysis showed that early response to RLT (i.e. DCR) detected in WBS is significantly associated with improved OS. Patients with PD had a significantly higher risk of death: HR 2.81 in the second cycle (**A**) and 2.38 in the third cycle (**B**). Similar results were found for patients with either DCR or PD in the PET after two cycles of RLT (**C**). Patients who experienced a PSA decline of at least 50% after two cycles of RLT also showed a significant improvement in OS (**D**)

Clinical follow-up data, as well as PSA and ALP responses, are available for all patients during and after completion of RLT or discontinuation due to progression. The median OS time for the entire cohort was 14.5 months (95%CI: 11.9–15.9). Kaplan-Meier analysis revealed a significant shorter median OS for patients who experienced PD in second cycle WBS (13 vs. 24 months, log-rank *P* < 0.001) with a HR of 2.81 (95%CI: 1.94–4.07, *P* < 0.001; Fig. 3A). Similar results were found for PD in the WBS of the third cycle (15 vs. 25 months, log-rank *P* < 0.001; Fig. 3B). The PET after two cycles of RLT revealed comparable findings (11 vs. 24 months, log-rank *P* < 0.001) with a HR of 3.5 (95%CI: 2.38–5.12, *P* < 0.001; Fig. 3C). Patients with a PSA decline of at least 50% had a significantly longer OS at various time points during treatment. Those with a PSA decline over 50% at the PET after two cycles of RLT had an improved OS of 26 vs. 17 months (log-rank *P* < 0.001) with a HR of 0.5 (95%CI: 0.33–0.76, *P* < 0.001; Fig. 3D).

At the end of the treatment, a significant association was found between PSA response and improved OS. Patients without a PSA-decline of 50% had a significantly longer OS with 27 vs. 14 months (log-rank *P* < 0.001; HR 0.41, 95%CI: 0.28 - 0.593, *P* < 0.001; Fig. 1S). Patients with any decline in ALP levels at the end of treatment had a significant longer OS (22 vs. 18 months, log-rank *P* = 0.03; HR 0.68, 95%CI: 0.47 - 0.97, *P* = 0.036; Fig. 1S).

## Discussion

This retrospective study analyzed data from 188 patients with mCRPC treated with a total of 898 cycles of RLT, focusing on the comparison on the prognostic value of WBS and interim PET scans. Our study demonstrated that OS can be prognosticated out of simple WBS after the second and third cycle of RLT, with comparable results and HRs as PSMA-PET after two cycles of RLT. The interim PET scan after 2 cycles of RLT showed a higher HR for the occurrence of death than WBS in the second and third cycles. This is probably due to its higher sensitivity and resolution. However, survival rates for patients with DCR were almost similar between these imaging modalities.

The median OS time of 14.5 months is consistent with the results of the VISION study, which reported a median OS time of 15.3 months [10]. Thus, prognosticating OS in this study population can validate therapy monitoring through response assessment of WBS and interim PET scans.

Several investigations have shown that serial PSMA-PET scans can reliably prognosticate OS in RLT and allow for treatment monitoring and decision support, based on the PSMA expression of the entire tumor burden [14, 25].

However, the use of PET scanners is limited by their availability, making it a resource-intensive and expensive option for clinical purposes. New generation SPECT/CT systems facilitate SPECT quantification. Nevertheless, whole-body SPECT is still time-consuming and resource intensive. Following our results WBS seem to be a reliable alternative to routine PET-based restaging after two cycles of RLT. Both imaging modalities, PET and WBS, can present misleading results, as decreasing PSMA-positive tumor volume can erroneously be interpreted as response but in fact is caused by dedifferentiation [26]. This fact is even more important to consider for WBS, as morphological imaging is not routinely acquired in post therapeutic imaging, in contrast to PET-CT or-MRI. This again underlines that assessing response should rely on multiple parameters, including imaging and PSA-value. As a prerequisite for RLT, the VISION study required PSMA-positive lesions greater than liver uptake [10]. Seifert et al. (2023) recently published PROMISE V2, an updated scoring system for evaluating PSMA-PET derived disease extend and PSMA expression in a standardized manner [16]. This enabled improved comparisons between different imaging modalities across various treatment cycles, promoting greater consistency among observers. To ensure effective implementation in the clinical setting, we compared the planar WBS with the MIP of the PET scan. In cases of advanced or disseminated disease, changes in PSMA expression can be detected with high reliability. Nevertheless, the evaluation is limited in cases of localized disease or due to physiological organ uptake, such as in the intestines or bladder. Small changes, in particular, may not be detected due to reduced resolution in the WBS. However, this study demonstrates a strong correlation between WBS and PET scan in terms of PSMA-expression, as the majority of patients had advanced disease with high tumor volume.

In this investigation, we also analyzed early and end of treatment PSA response with the established cut-offs (PSA50). Hence, similar survival rates were found [17]. In this mCRPC group a PSA decline of at least 50% at the PET after 2 cycles of RLT timepoint was prognostic in predicting OS (26 vs. 17 months). These findings are consistent with previous research [10, 20]. A comparison of PSA50 responses revealed a correlation that was intermediate but nevertheless significant in comparison to treatment responses in WBS of the third cycle and the PET after 2 cycles. This is likely attributable to the high cut-off of 50%, which may have resulted in the treatment effect and changes in biochemical response exhibiting considerable heterogeneity in mCRPC, particularly given the influence of tumor volume. Heinrich et al. (2018) stated in a review that ALP can serve as a reliable prognostic marker in various retrospective analyses and should therefore be monitored [27]. Our study found a trend suggesting that patients who experienced a decline in ALP at the end of treatment had a slightly higher OS (22 vs 18 months). Therefore, changes in ALP levels may indicate a response in bone metastatic disease in mCRPC, as previously reported [28]. However, it is important to note that changes in ALP may only have a small impact on OS and should not be the sole variable used to assess response to treatment.

In line with proceedings in prospective VISION trial and based on the findings of this investigation restaging PSMA-PET after two therapy cycles is not performed in all patients, but based on individual case decisions, if appropriate. Patients are now regularly observed using post-therapeutic WBS and PSA values to assess treatment response.

This single center investigation is limited by its retrospective approach. This results in very different number of cycles of RLT per patient as the decision of discontinuation of therapy was made on an individual basis, considering all available clinical and imaging-based parameters. The imaging modalities were evaluated solely using a visual scale. While this approach is similar to clinical routine, it may lead to misjudgments, particularly in patients with small tumor volumes. One drawback of using WBS for response assessment is that this imaging modality is directly connected to the application of another therapeutic dose. Therefore PSMA-PET corroborates its important role for response assessment after, or in case of suspected progression also under RLT. The OS time may be subject to selection bias due to the exclusion of patients who received therapy but did not undergo first interim PET after 2 cycles.

## Conclusion

In this study population, the assessment of early treatment response with WBS of [^177^Lu]PSMA-617 therapy appears to have a prognostic value. Therefore, it does not seem necessary to conduct additional PSMA-PET imaging to evaluate the success of ongoing RLT during the first four cycles of therapy.

## Electronic supplementary material

Below is the link to the electronic supplementary material.

Supplementary Material 1

## Data Availability

The datasets generated during and/or analysed during the current study are available from the corresponding author on reasonable request.

## List of abbreviations

PC: prostate cancer
mCRPC: metastatic castration resistant prostate cancer
OS: overall survival.
RLT: radioligand therapy
PSMA: prostate specific membrane antigen
PFS: progression-free survival
PET: positron emission tomography
PCWG3: the prostate cancer working group criteria 3
CT: computed tomography
PSA: prostate specific antigen
WBS: whole-body-scan.
SPECT: single photon emission computed tomography
MRI: magnetic resonance imaging.
CR: complete response
PR: partial response
SD: stable disease
PD: progressive disease
DCR: disease control rate.
HR: hazard ratio.
ALP: alkaline phosphatase.

## References

[1] Sung H, Ferlay J, Siegel RL, Laversanne M, Soerjomataram I, Jemal A, Bray F. Global Cancer statistics 2020: GLOBOCAN estimates of incidence and Mortality Worldwide for 36 cancers in 185 countries. CA Cancer J Clin. 2021;71:209–49. 10.3322/CAAC.21660. 33538338 10.3322/caac.21660

[2] Sandhu S, Moore CM, Chiong E, Beltran H, Bristow RG, Williams SG. Prostate Cancer. Lancet. 2021. 10.1016/S0140-6736(21)00950-8. 398;1075–1090. 34370973 10.1016/S0140-6736(21)00950-8

[3] Armstrong AJ, Szmulewitz RZ, Petrylak DP, Holzbeierlein J, Villers A, Azad A, et al. ARCHES: a Randomized, Phase III Study of Androgen Deprivation Therapy with Enzalutamide or Placebo in Men with metastatic hormone-sensitive prostate Cancer. J Clin Oncol. 2019;37:2974–86. 10.1200/JCO.19.00799. 31329516 10.1200/JCO.19.00799 PMC6839905

[4] Sartor O, de Bono JS. Metastatic prostate Cancer. N Engl J Med. 2018;378:645–57. 10.1056/NEJMRA1701695. 29412780 10.1056/NEJMra1701695

[5] Gillessen S, Armstrong A, Attard G, Beer TM, Beltran H, Bjartell A et al. Management of Patients with Advanced Prostate Cancer: Report from the Advanced Prostate Cancer Consensus Conference 2021. Eur Urol. 2022;82:115–141. 10.1016/J.EURURO.2022.04.002 10.1016/j.eururo.2022.04.002 35450732

[6] de Bono J, Mateo J, Fizazi K, Saad F, Shore N, Sandhu S, et al. Olaparib for metastatic castration-resistant prostate Cancer. N Engl J Med. 2020;382:2091–102. 10.1056/NEJMOA1911440. 32343890 10.1056/NEJMoa1911440

[7] Antonarakis ES, Piulats JM, Gross-Goupil M, Goh J, Ojamaa K, Hoimes CJ, et al. Pembrolizumab for treatment-refractory metastatic castration-resistant prostate Cancer: Multicohort, open-label phase II KEYNOTE-199 study. J Clin Oncol. 2020;38:395–405. 10.1200/JCO.19.01638. 31774688 10.1200/JCO.19.01638 PMC7186583

[8] Rahbar K, Essler M, Pabst KM, Eiber M, la Fougère C, Prasad V, et al. Safety and survival outcomes of 177Lu-Prostate-specific membrane Antigen Therapy in patients with metastatic castration-resistant prostate Cancer with prior 223Ra treatment: the RALU Study. J Nucl Med. 2023;64:574. 10.2967/JNUMED.122.264456. 36302656 10.2967/jnumed.122.264456 PMC10071785

[9] Parker C, Nilsson S, Heinrich D, Helle SI, O’Sullivan JM, Fosså SD, et al. Alpha Emitter Radium-223 and survival in metastatic prostate Cancer. N Engl J Med. 2013;369:213–23. 10.1056/NEJMOA1213755. 23863050 10.1056/NEJMoa1213755

[10] Sartor O, de Bono J, Chi KN, Fizazi K, Herrmann K, Rahbar K, et al. Lutetium-177–PSMA-617 for metastatic castration-resistant prostate Cancer. N Engl J Med. 2021;385:1091. 10.1056/NEJMOA2107322. 34161051 10.1056/NEJMoa2107322 PMC8446332

[11] Hoffman A, Amiel GE. The impact of PSMA PET/CT on modern prostate Cancer Management and decision making-the Urological Perspective. Cancers (Basel). 2023;15. 10.3390/CANCERS15133402. 10.3390/cancers15133402 PMC10340269 37444512

[12] Sprute K, Kramer V, Koerber SA, Meneses M, Fernandez R, Soza-Ried C, et al. Diagnostic accuracy of 18F-PSMA-1007 PET/CT Imaging for Lymph Node Staging of Prostate Carcinoma in primary and biochemical recurrence. J Nucl Med. 2021;62:208–13. 10.2967/JNUMED.120.246363. 32817141 10.2967/jnumed.120.246363 PMC8679593

[13] Murthy V, Sonni I, Jariwala N, Juarez R, Reiter RE, Raman SS, Hope TA. The role of PSMA PET/CT and PET/MRI in the initial staging of prostate Cancer. Eur Urol Focus. 2021;7:258–66. 10.1016/J.EUF.2021.01.016. 33541838 10.1016/j.euf.2021.01.016

[14] Seifert R, Alberts IL, Afshar-Oromieh A, Rahbar K. Prostate Cancer Theranostics: PSMA targeted therapy. PET Clin. 2021;16:391–6. 10.1016/J.CPET.2021.03.004. 34053583 10.1016/j.cpet.2021.03.004

[15] Kratochwil C, Fendler WP, Eiber M, Hofman MS, Emmett L, Calais J, et al. Joint EANM/SNMMI Procedure Guideline for the use of 177Lu-Labeled PSMA-Targeted Radioligand-Therapy (177Lu-PSMA-RLT). Eur J Nucl Med Mol Imaging. 2023;50:2830–45. 10.1007/S00259-023-06255-8. 37246997 10.1007/s00259-023-06255-8 PMC10317889

[16] Seifert R, Emmett L, Rowe SP, Herrmann K, Hadaschik B, Calais J, et al. Second Version of the prostate Cancer molecular imaging standardized evaluation Framework including response evaluation for clinical trials (PROMISE V2). Eur Urol. 2023;83:405–12. 10.1016/J.EURURO.2023.02.002. 36935345 10.1016/j.eururo.2023.02.002

[17] Scher HI, Morris MJ, Stadler WM, Higano C, Basch E, Fizazi K, et al. Trial Design and objectives for castration-resistant prostate Cancer: updated recommendations from the prostate Cancer clinical trials Working Group 3. J Clin Oncol. 2016;34:1402–18. 10.1200/JCO.2015.64.2702. 26903579 10.1200/JCO.2015.64.2702 PMC4872347

[18] Pathmanandavel S, Crumbaker M, Ho B, Yam AO, Wilson P, Niman R, et al. Evaluation of 177Lu-PSMA-617 SPECT/CT Quantitation as a response Biomarker within a prospective 177Lu-PSMA-617 and NOX66 combination trial (LuPIN). J Nucl Med. 2023;64:221–6. 10.2967/JNUMED.122.264398. 36008120 10.2967/jnumed.122.264398 PMC9902857

[19] John N, Pathmanandavel S, Crumbaker M, Counter W, Ho B, Yam AO, et al. 177Lu-PSMA SPECT quantitation at 6 weeks (dose 2) predicts short progression-free survival for patients undergoing 177Lu-PSMA-I&T therapy. J Nucl Med. 2023;64:410–5. 10.2967/JNUMED.122.264677. 36215568 10.2967/jnumed.122.264677

[20] Neubauer MC, Nicolas GP, Bauman A, Fani M, Nitzsche E, Afshar-Oromieh A, et al. Early response monitoring during [177Lu]Lu-PSMA I&T therapy with quantitated SPECT/CT Predicts Overall Survival of MCRPC patients: Subgroup Analysis of a swiss-wide prospective Registry Study. Eur J Nucl Med Mol Imaging. 2023. 10.1007/S00259-023-06536-2. 38038755 10.1007/s00259-023-06536-2 PMC10881597

[21] Rassek P, Schäfers M, Rahbar K, Backhaus P. [18F]-PSMA-1007-PET for evaluation of kidney function. Nuklearmedizin. 2023;62:244–51. 10.1055/A-2127-7880. 37595624 10.1055/a-2127-7880

[22] Seifert R, Rasul S, Seitzer K, Eveslage M, Nikoukar LR, Kessel K, et al. A prognostic risk score for prostate Cancer based on PSMA PET–Derived organ-specific Tumor volumes. Radiology. 2023;307(4):e222010. 10.1148/radiol.222010. 37070991 10.1148/radiol.222010 PMC10838189

[23] Hofman MS, Emmett L, Violet JY, Zhang A, Lawrence NJ, Stockler M, et al. TheraP: a randomized phase 2 trial of 177 Lu-PSMA-617 theranostic treatment vs Cabazitaxel in Progressive Metastatic Castration-resistant prostate Cancer (Clinical Trial Protocol ANZUP 1603). BJU Int. 2019;124:5–13. 10.1111/BJU.14876. 31638341 10.1111/bju.14876

[24] Rahbar K, Ahmadzadehfar H, Kratochwil C, Haberkorn U, Schafers M, Essler M, et al. German Multicenter Study investigating 177Lu-PSMA-617 Radioligand therapy in advanced prostate Cancer patients. J Nucl Med. 2017;58:85–90. 10.2967/JNUMED.116.183194. 27765862 10.2967/jnumed.116.183194

[25] Burgard C, Hein C, Blickle A, Bartholomä M, Maus S, Petto S, et al. Change in total lesion PSMA (TLP) during [177Lu]Lu-PSMA-617 Radioligand Therapy predicts overall survival in patients with MCRPC: monocentric evaluation of a prospective Registry. Eur J Nucl Med Mol Imaging. 2023. 10.1007/S00259-023-06476-X. 37889298 10.1007/s00259-023-06476-x PMC10796576

[26] Seifert R, Kessel K, Schlack K, Weckesser M, Kersting D, Seitzer KE, et al. Total tumor volume reduction and low PSMA expression in patients receiving Lu-PSMA Therapy. Theranostics. 2021;11:8143–51. 10.7150/THNO.60222. 34373733 10.7150/thno.60222 PMC8344008

[27] Heinrich D, Bruland O, Guise TA, Suzuki H, Sartor O. Alkaline phosphatase in metastatic castration-resistant prostate Cancer: reassessment of an older biomarker. Future Oncol. 2018;14(24):2543–56. 10.2217/fon-2018-0087. 29925281 10.2217/fon-2018-0087

[28] Scalzi P, Baiocco C, Genovese S, Trevisan A, Sirotova Z, Poti C. Evaluation of bone metastases by 18F-Choline PET/CT in a patient with castration-resistant prostate Cancer treated with Radium-223. Urologia. 2017;84:61–4. 10.5301/uro.5000206. 27911459 10.5301/uro.5000206

