# Supplementary material for: Early treatment response assessment with [177Lu]PSMA whole-body-scintigraphy compared to interim PSMA-PET
Source: Cancer Imaging. 2024 Sep 19;24:126. doi: 10.1186/s40644-024-00773-w (PMC11414098; doi:10.1186/s40644-024-00773-w)

**Table 1S:** Lowest and highest lesional PROMISE V2 expression score

| **Promise V2 expression score of the lowest lesions** | Initial PET  (n = 188) | First Cycle  (n = 188) | Second Cycle  (n = 188) | Interim PET  (n = 188) | Third Cycle  (n = 149) |
| --- | --- | --- | --- | --- | --- |
| **0** | 0 | 0 | 0 | 0 | 0 |
| **1** | 7 (3.7) | 10 (5.3) | 11 (5.9) | 15 (8.0) | 14 (9.4) |
| **2** | 135 (71.8) | 133 (70.7) | 130 (69.1) | 108 (57.4) | 87 (58.4) |
| **3** | 46 (24.5) | 45 (23.9) | 47 (25.0) | 65 (34.6) | 48 (32.2) |

| **Promise V2 expression score of the highest lesions** | Initial PET  (n = 188) | First Cycle  (n = 188) | Second Cycle  (n = 188) | Interim PET  (n = 188) | Third Cycle  (n = 149) |
| --- | --- | --- | --- | --- | --- |
| **0** | 0 | 0 | 0 | 0 | 0 |
| **1** | 0 | 0 | 0 | 1 (0.5) | 2 (1.3) |
| **2** | 7 (3.7) | 5 (2.7) | 9 (4.8) | 14 (7.4) | 10 (6.7) |
| **3** | 181 (96.3) | 183 (97.3) | 179 (95.2) | 173 (92.0) | 137 (72.9) |

**Figure 1S:** Overall survival analysis


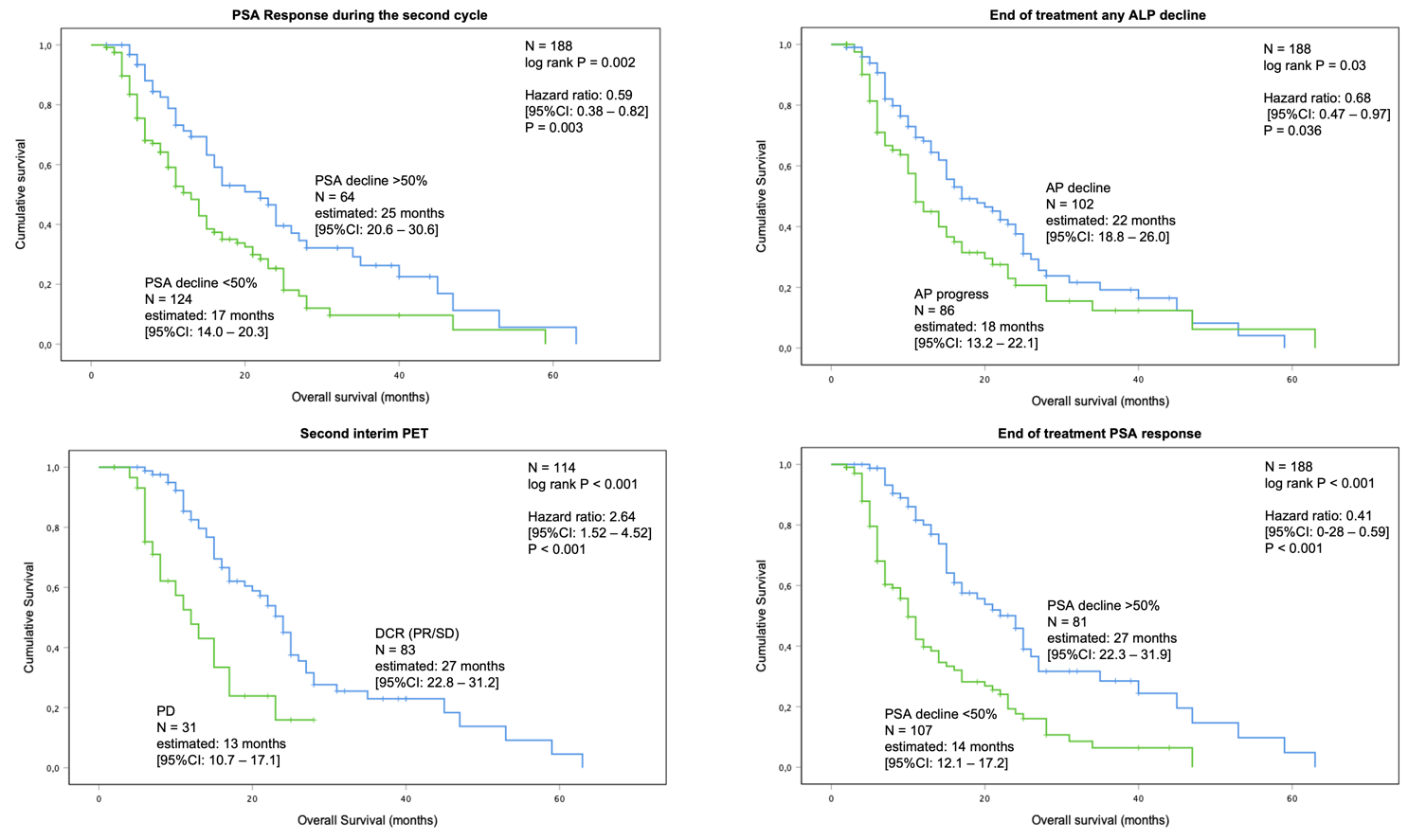

Supplement: Supplementary file 1 — Supplementary Material 1 [file 40644_2024_773_MOESM1_ESM.docx]
